# Revealing the secrets of a 2900-year-old clay brick, discovering a time capsule of ancient DNA

**DOI:** 10.1038/s41598-023-38191-w

**Published:** 2023-08-22

**Authors:** Troels Pank Arbøll, Sophie Lund Rasmussen, Nadieh de Jonge, Anne Haslund Hansen, Cino Pertoldi, Jeppe Lund Nielsen

**Affiliations:** 1 Department of Cross-Cultural and Regional Studies, University of Copenhagen, Copenhagen, Denmark; 2 Faculty of Asian and Middle Eastern Studies, University of Oxford, Oxford, UK; 3 Linacre College, Oxford, UK; 4 Wildlife Conservation Research Unit, The Recanati-Kaplan Centre, Department of Biology, University of Oxford, Abingdon, UK; 5 Department of Chemistry and Bioscience, Aalborg University, Aalborg, Denmark; 6 Modern History and World Cultures, National Museum of Denmark, Copenhagen, Denmark; 7 Aalborg Zoo, Aalborg, Denmark

**Keywords:** Genetics, Agricultural genetics, Evolutionary ecology, Palaeoecology

## Abstract

The recent development of techniques to sequence ancient DNA has provided valuable insights into the civilisations that came before us. However, the full potential of these methods has yet to be realised. We extracted ancient DNA from a recently exposed fracture surface of a clay brick deriving from the palace of king Ashurnasirpal II (883–859 BCE) in Nimrud, Iraq. We detected 34 unique taxonomic groups of plants. With this research we have made the pioneering discovery that ancient DNA, effectively protected from contamination inside a mass of clay, can successfully be extracted from a 2900-year-old clay brick. We encourage future research into this subject, as the scientific prospects for this approach are substantial, potentially leading to a deeper understanding of ancient and lost civilisations.

## Introduction

Near the river Tigris, outside the ancient city of Kalhu, known today as Nimrud, a brickmaker once prepared a clay brick for the construction of a new palace dedicated to his king Ashurnasirpal II (approximately 883–859 BCE). Little did he know, that almost 2900 years later, this insignificant clay brick would serve as a unique time capsule revealing details of the flora from this specific area and time, through the modern-day investigation of the ancient DNA hidden and preserved for thousands of years.

### The sampling material

**Figure 1:**
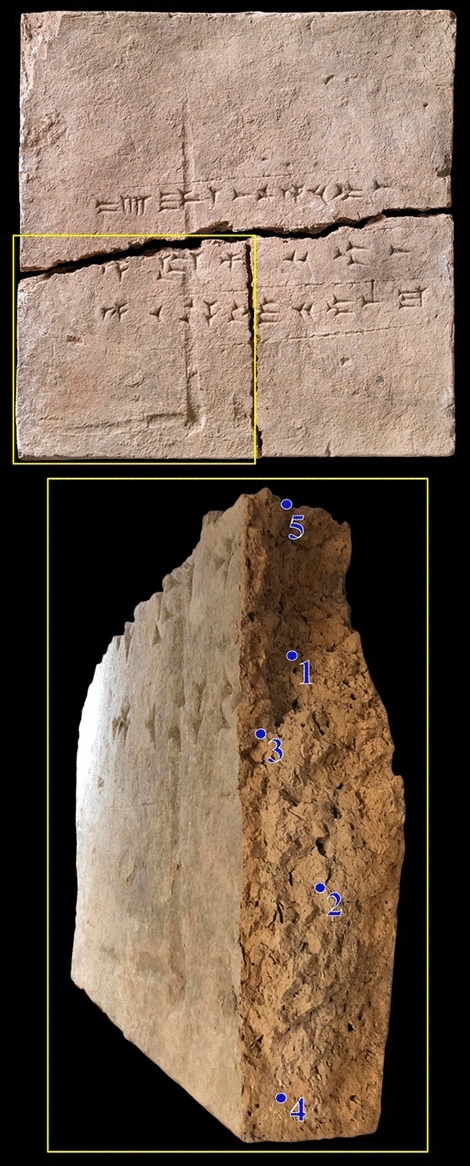
The clay brick from which the samples derived. Pictures of the clay brick from the National Museum of Denmark (museum number 13854) and the five sampling points on the surface of the break. The yellow square in the upper part of the figure represents the piece of the brick illustrated below.

This investigation presents the discovery of ancient DNA (aDNA) in samples from an approximately 2900-year-old clay brick kept at the National Museum of Denmark. Showcasing a novel application of aDNA analysis and its results, we provide a discussion of the identified flora in relation to the rich abundance of textual evidence available from ancient Mesopotamia (roughly modern-day Iraq and Syria) to situate our findings in broader current discussions regarding the domestication of plants in this area. The brick in question (museum number 13854) was donated to the National Museum of Denmark in 1958 by the Rask Ørsted Foundation. It was discovered during the British excavations of Nimrud, beginning in 1949. Assyriologist Jørgen Læssøe ensured financial support from the state-funded Rask Ørsted Foundation making it possible for him and other Danes to participate in the excavations. In 1958, the National Museum received a group of objects from Nimrud, including the brick in question, in acknowledgement of the support. At the time when it entered the collection at the National Museum of Denmark, it had already broken into two pieces horizontally. Due to their state, mudbricks are seemingly solid, yet delicate in nature. During an otherwise controlled handling in 2020, the lower half of the brick unfortunately split vertically into two pieces. This event presented an opportunity for a scientific study of uncontaminated clay that could be dated with relative certainty. It was from this new uncontaminated break that the samples for this study were extracted (Fig. 1).

Made primarily of mud collected locally near the Tigris river, mixed with some botanical material such as chaff or straw, or animal dung, the brick was shaped in a mould before it was inscribed with so-called cuneiform signs, recording a dialect of the now extinct Semitic language Akkadian, after which it was placed in the sun to dry^1,2^. Mudbricks with cuneiform inscriptions were traditionally used for the construction of monumental buildings by rulers in ancient Mesopotamia from the late 3^rd^ through the late 1^st^ millennia BCE, and thousands of these bricks are known today. It is possible to date this specific brick within a decade due to its inscription, which identifies it as: “The property of the palace of Ashurnasirpal, king of Assyria”. The text also provides the king with a genealogy securely placing him chronologically in a list of known rulers. The construction of Ashurnasirpal II’s palace at ancient Kalhu (modern-day Nimrud)—known today as the North-West palace—celebrated the city as the new capital of Assyria at the dawn of the Neo-Assyrian Empire, and its construction was begun around 879 BCE^3^. The absolute chronology of the Neo-Assyrian period (ca. 883–612 BCE) is in large parts well established and based on surviving lists on cuneiform clay tablets, from which it is possible to correlate years in a king’s reign with the name of a specific official in his government^4^. These data are linked to astronomical events recorded on specific dates in especially the seventh century BCE^5^. The data we obtained from this brick presented an entirely new opportunity to study different aspects of the ninth century BCE.

### The history of aDNA research

The first use of ancient DNA (aDNA) dates back to 1984, where dried muscle tissue from an extinct species was sequenced from museum specimens for the first time^6^. Since then, aDNA studies have allowed for a unique look back in time, into the genomic content of extinct animal species, humans, as well as archaeozoological and archaeobotanical studies from various preserved specimens^7^. Our study represents a new material from which aDNA can be analysed in the form of a clay brick. Ancient DNA is difficult to extract due to its low concentration and high degree of fragmentation, but in this study, it was possible to analyse the aDNA with a direct link to context and date, which allowed us to correlate with roughly contemporary textual evidence to present living plants.

### Nimrud—the historical site represented

The city of Kalhu has held a prominent place in the study of the ancient Near East due to its excavation history. From 1845, Sir Austen Henry Layard conducted the first excavations at the site, and it was only the second ancient Mesopotamian city to be unearthed at the time. The findings formed the basis for much of the initial knowledge about the so-called “Cradle of Civilisation” that shaped the subject Assyriology. The site was explored periodically after Layard’s fieldwork, e.g., from 1949 and onwards by the prominent British archaeologist Max Mallowan alongside his wife, and famed author, Agatha Christie. Within the past decade, the remains of the North-West palace have been partially destroyed due to the recent upheaval in northern Iraq and large parts of Syria.

## Results

Through extraction and sequencing of aDNA from the clay brick (see Materials and Methods) and the following data analysis, we were able to detect 34 unique taxonomic groups of plants representing the order Laurales as well as seven distinct families from other orders: Apiaceae (subfamily *Apioideae*, tribe Selineae), Betulaceae, Brassicaceae (including the genus *Brassica*), Ericaceae (including the subfamilies *Ericoidae* and *Vaccinioideae*), Poaceae (tribe Poeae and Triticeae), Fagaceae (genus *Quercus*), and Salicaceae (Table 1).

**Table 1:** Overview of the unique taxonomic groups (OTUs) of plants detected in the samples.

Sample number	Apiodeae sp.	Betulaceae sp.	Brassicaeae sp.	*Brassica* sp.	Ericaceae sp.	*Ericoidae* sp.	*Vaccinioideae* sp.	Laurales sp.	*Quercus* sp.	Salicaceae sp.	Selineae sp.	Poeae sp.	Triticeae sp.
1	1 OTU	ND	ND	ND	ND	ND	ND	ND	ND	ND	3 OTUs	ND	ND
2	ND	ND	8 OTUs	1 OTU	1 OTU	ND	ND	ND	ND	ND	ND	ND	ND
3	ND	ND	ND	ND	ND	ND	ND	ND	ND	ND	ND	ND	ND
4	ND	ND	ND	ND	3 OTUs	5 OTUs	ND	1 OTU	1 OTU	1 OTU	1 OTU	ND	1 OTU
5	1 OTU	2 OTUs	8 OTUs	1 OTU	4 OTUs	5 OTUs	1 OTU	2 OTUs	1 OTU	1 OTU	1 OTU	2 OTUs	3 OTUs

The identified plants are grouped by taxonomic relationship, and the number of uniquely observed OTUs of each taxonomic group per sample is indicated. Classification of the identified plants were generally limited to the family level, or even above, in order to minimise the chance of reporting false positive identifications from short sequences.

*ND* not detected.

The largest number of identifications were made from sample 5, followed by samples 2 and 4, while sample 3 was discarded as no satisfactory sequencing result could be obtained. The most abundant sequences of plants were from the families Brassicaceae (cabbage) and Ericaceae (heather). Furthermore, contributions were observed from the families Betulaceae (birch), Lauraceae (laurels), Selineae (umbellifiers) and Triticeae (cultivated grasses).

Control samples from the workbenches used using DNA extraction, negative DNA extraction sample, as well as negative PCR controls did not yield amplification of plant DNA, nor sequencing reads after inclusion in the Nanopore sequencing run.

All samples were also analysed with a short DNA barcode targeting vertebrates to identify potential sources of modern DNA contamination. From these analyses, the primary sources identified were human and swine, both of which were observed sporadically across all samples. Based on this observation, results from this DNA barcode were not analysed further, due to the likelihood of modern DNA traces obscuring potential aDNA results.

Furthermore, the remaining DNA extracts from samples 2 and 5 were prepared for metagenomic sequencing to attempt to verify the results as ancient DNA traces, by investigating the characteristic deamination damages in the ends of aDNA molecules. However, it was not possible to obtain enough high-quality reads to reliably perform this analysis. Manual curation of the 105.000 reads obtained after 16 h of Nanopore sequencing revealed that approx. 0.1% of the reads obtained could be mapped to plant species belonging to the taxonomic groups observed with the amplicon sequencing, with relatively high statistical strength, due to their short length (30–140 bp).

## Discussion

Previous analyses of the flora of modern-day Iraq show >3300 species in 908 genera belonging to 136 families of flowering plants^8^.

The aDNA screening showed plant species from seven distinct families, excluding the findings from the order Laurales, as these sequences could not confidently be defined at family level. We acknowledge that some of the observed taxonomic groups of plants have a wide distribution across the planet, which is a limitation of our study given that no direct evidence of ancient DNA traces could be generated. However, stringent controls and data treatment strongly suggest that the obtained DNA sequences from plants originated from the clay brick, and we will discuss the results in line with this. These include Apiaceae, a family of herbs with over 200 genera and 3000 species^9^, out of which 155 species are represented in modern-day Iraq including seven endemic species^8^. We detected sequences of the tribe Selineae, belonging to the family Apiaceae, in our samples. A range of these species found in Iraq are food plants such as *Daucus* (carrot), *Pastinaca* (parsnip), *Apium* (celery), some containing lethal alkaloid poisons (*Conium* spp.), whereas others are used for cooking, as well as modern-day medicinal purposes (*Carum, Foeniculum, Pimpinella, Anethum* spp.).

The birch family, Betulaceae, is a family of deciduous trees and shrubs consisting of six genera^10^ with only one genus, *Betula*, and one species, *Betula pendula*, found in Iraq today^11^. We detected DNA sequences from the birch family, Betulaceae, in our samples. With only one species, *Betula pendula*, represented in the botanical composition of modern-day Iraq^11^, and assuming that the passing of 2900 years has not caused a reduction in the number of species belonging to the genus *Betula* in Iraq, we may suggest an identification of the species *Betula pendula* in our samples.

The family Salicaceae contains dioecious deciduous trees and shrubs of soft, light wood, and comprises three-four genera and over 500 species, with two genera, *Salix* (willow) and *Populus* (poplar), represented in modern-day Iraq^11^.

The Fagaceae family consists of deciduous or evergreen trees or shrubs with simple, alternate leaves and has six genera and over 800 species, out of which only four species from one genus, *Quercus* (oak), is present in Iraq today^11^. Therefore, we allude that our samples could have contained one or more of the four following species: *Q. libani*, *Q. infectoria*, *Q. macranthera*, and *Q. aegilops*^8,11^.

The mustard or cabbage family, or Brassicaceae (Cruciferae), contains 338 genera and 3709 species^12^, and is represented with 79 genera and 195 species including ten endemic species in modern-day Iraq^8^. We identified DNA sequences from the genus *Brassica* in the samples. *Brassica* includes vegetables such as cabbage, kale, broccoli, cauliflower, root and stem crops like radish, horseradish, turnips, kohlrabi, and seed crops such as mustard seed and rapeseed^13^. Eight species belonging to *Brassica* (four native and four cultivated) can be found in modern-day Iraq: *B. deflexa, B. nigra, B. juncea, B. tournefortii,* as well as *B. oleracea, B. rapa, B. napus,* and *B. elongata*^14^.

The family Poaceae (grass family) includes 11,500 species^15^, out of which 101 genera with a total of 264 species including three endemic species can be found in Iraq today^8^. We identified DNA sequences from the tribes Poeae and Triticeae in our samples. The genus *Poa* belonging to the tribe Poeae with 11–12 species represented in modern-day Iraq^16^. The tribe Triticeae contains several of the important cereal grasses such as wheat, rye and barley. In modern-day Iraq 12 genera of the Triticeae tribe are represented, such as *Tricitum* (wheat), *Hordeum* (barley) and *Secale* (rye)^16^.

The heath family, Ericaceae, with > 100 genera and > 4400 species, is a very diverse family with life forms ranging from trees to epiphytes and small shrubs to herbs without chlorophyll^17,18^. We identified DNA sequences affiliating with the subfamilies *Ericoidae* and *Vaccinioideae* in our samples.

Laurales, an order of mostly tropical trees or shrubs, often with scented wood of great durability, is comprised of seven families that contain 100 genera with approximately 2500–2800 species^19^. The seven families are Atherospermataceae, Calycanthaceae, Gomortegaceae, Hernandiaceae, Lauraceae, Monimiaceae and Siparunaceae^19^.

The study of ancient Mesopotamia, “the Cradle of Civilisation”, is based primarily on the investigation of written sources and archaeological material. Researchers have a substantial corpus of written sources available, consisting of tens of thousands of clay tablets inscribed with cuneiform script recording the ancient Akkadian and Sumerian languages from ca. 3200 BCE–75 CE. Compared to other archaeological materials from the same time periods, the clay tablets remain extraordinarily well preserved. However, it has so far been challenging to identify the plant species described in ancient cuneiform literature, as the terms and concepts differ from the species’ names applied by modern-day science. Furthermore, we only have limited visual depictions of ancient flora and fauna. This gap in knowledge is problematic for the study of ancient medicine, as we know the ancient names of many pharmaceutical ingredients from the cuneiform texts, but have yet to identify them correctly in a modern context. By introducing analysis of aDNA as a supplementary tool for the process of identifying species present in the ancient Mesopotamian civilisations, this field of research could develop considerably in the coming years. Furthermore, future explorations of the method presented here in neighboring civilisations would enable similar inquiries into other ancient disciplines.

A multitude of terms for ancient Iraqi flora have been passed down to us. Especially texts from the second and first millennia BCE are useful for discussing our data in relation to ancient terminology, as these writings provide us with much knowledge of agricultural products as well as ingredients used in medical prescriptions, pharmaceutical mixtures, and various rituals. Particularly, two texts offer useful evidence for a wide variety of garden plants, namely a list of plants imported and various items served during Ashurnasirpal II’s inaugural festivities in relation to his palace, as well as a list of plants from the garden of the Babylonian king Marduk-apla-iddina (Biblical Merodach-Baladan) in the eighth century BCE^20,21^.

Previous work correlating modern terms for plants with ancient words has focused primarily on identifying cognate words in Arabic, Aramaic, and Hebrew^22^, though this approach holds limited potential for linking ancient terminology with exact biological species. This investigation provides a partial solution to overcoming the problems inherent in attempting to identify concrete taxonomic groups of plants through linguistics. Our study identifies the presence of specific families of ancient flora in a confined region during a limited time period, although it does not provide ancient terms for these findings. In the following we discuss our most relevant results and assign them to Akkadian terms for ancient flora to the best of our ability with the current state of knowledge. At the same time, we offer a review of the impact of our findings on the history of domestication of specific plants.

Interestingly, a range of the taxonomic groups of flora identified in our study are uncommon in the cuneiform documentation, likely because they were not grown or circulated on a wider scale in the main administrative centres of the first millennium BCE employing cuneiform writing. Thus, our results may not be representative of the bureaucratic focuses of the administrative units or temples of the time^23^, in case the plants did not form part of a diet or crop cultivation under central administration. Furthermore, it is to be expected that some ancient plant taxonomies differ from modern biological classification. In ancient times, Ashurnasirpal II established a sort of zoological garden at Nimrud with exotic animals, and he also imported numerous plants from regions throughout the Middle East to reshape the agricultural landscape of the region^21,24,25^. However, his initiatives may not have been implemented properly before the palace was completed. It therefore remains plausible that our findings represent local flora from the Nimrud region in the ninth century BCE, even though some of the plant families were not represented in the administrative literature from the period.

We identified nine taxonomic groups related to the cabbage family Brassicaceae, of which one could be assigned to the genus *Brassica*. The exact time and place of the domestication of cabbage remains unclear, though a recent study suggests it may have occurred in the north-central and north-eastern Mediterranean areas in the first half of the first millennium BCE^26^. This conclusion was based on the absence of evidence, seeing as textual sources and depictions of cabbage, kale, and cole crops from the ancient Near East have largely eluded us^26^. We know that various types of greens, generally identified as lettuce, were grown and consumed in first millennium BCE Iraq^27^. It is noteworthy that several types of kale, wild and domesticated, are similar in appearance to lettuce (e.g., *Brassica oleracea*). Because one or more terms designating cabbage, kale, and cole crops appear to be lacking in Akkadian, though our results indicate that such species were present in northern Iraq in the ninth century BCE, it seems likely that we should seek terms for this type of green among the terminology for lettuce. One example includes the plant called ḪI.IZ.TUR in Sumerian, which was equated with the Akkadian greens *guzāzu* and *murāru* in lexical lists. The name of the latter vegetable comes from the word “bitter”, which has been tentatively translated as “bitter lettuce”. However, the only certainty is that these plants were greens that could be grown and consumed. On the basis of our findings, one might propose to correlate the bitter taste of cabbage with the name *murāru*. Thus, the claim that ancient cuneiform texts do not include cabbage, kale, or cole crops should perhaps be modified, seeing as they do in fact list types of lettuce, such as *murāru*, which could also be identified as a type of cabbage^28^.

Another category of plants represented in the genus *Brassica* is mustard. The Akkadian term *kasû* (Sumerian GAZI^sar^) has been interpreted as “mustard”^29^. According to the written sources it was grown in large quantities in various periods, and the plant as well as its products could be used as a flavouring agent. However, descriptions of the appearance of the plant do not precisely match those of the native mustard plant species known from Iraq. Accordingly, several researchers prefer to identify the plant as, e.g., beet, dodder, wild liquorice, or tamarind, as discussed recently by Eypper^30^. Still, none of the alternatives fit the descriptions of *kasû* perfectly, and it cannot be dismissed that descriptions of the *kasû*-plant’s appearance might fit a species of mustard native to Iraq.

A species such as radish (*Raphanus sativus*), belonging to the Brassicaceae family detected in our samples, is not widely attested in the cuneiform documentation, though a word likely identified as a form of radish, *puglu*, is applied in letters and administrative documents from the second half of the third millennium BCE and in a few texts from the first millennium BCE, among these at least one medical text^27,31^.

The identification of several DNA sequences belonging to the cabbage family (Brassicaceae) in the clay brick under investigation (Fig. 1), suggests that the theoretical conclusion by Maggioni et al.^26^, placing the origin of cole crops in the northern Mediterranean, may need to be modified^32^. Though such crops may have originated there, they were surely present in ninth century BCE northern Iraq, which is earlier than the primarily linguistic evidence presented by Maggioni et al.^26^. Whether our findings represent wild plants or domesticated plants growing on the banks of the Tigris remains uncertain.

The identification of aDNA in our samples from the family Poaceae, tribe Triticeae, could be indicative of the presence of a species such as *Triticum aestivum*. Bread wheat (*Triticum aestivum*) has a history of domestication reaching back at least 10,000 years^33^. Through the cultivation of other wheat species, such as emmer and einkorn, the plant was domesticated for thousands of years before the invention of writing in ancient Iraq^34^. In the Akkadian language wheat was called *kibtu* (Sumerian ŠE.GIG), though in one period it may have been known as *aršātu*. Grains of emmer and bread wheat were discovered at contemporary Nimrud^35^. Wheat was produced since the invention of writing, and we continuously find references in the texts to grain and various methods of production^36^. The heartland of ancient Assyria—including Nimrud—in the northern part of modern-day Iraq, is located in a climatic zone that could periodically sustain rainfed cultivation. Yet, as recent research has shown, the heartland of ancient Assyria experienced elevated levels of precipitation at the time of Ashurnasirpal II^37^, which could, in principle, have provided more favourable conditions for growing bread wheat and associated crops.

This research demonstrates that extracting aDNA from a clay brick enables the detection of a range of taxonomic groups present at the given time and location when the brick was made. The clay brick serves as a time capsule providing a unique insight into the biodiversity of a specific time and location.

It is rather certain that the clay used to create the brick analysed in the present study dates from around 879 BCE due to the inscription on the brick, and that the clay must have been gathered in the area surrounding the city of Nimrud. This information was made available in the historical sources explaining how the construction of the palace was initiated around 879 BCE as well as descriptions of the general practice of mudbrick creation. What is unknown is how the detected DNA ended up in this particular mass of clay. This challenges the interpretation of the results, as it is merely possible to describe the presence/absence of species with our method. However, when the data is combined and interpreted together with the knowledge gained from the numerous written contemporary historical sources, the information provided by this method, is very applicable and constitutes an important contribution to the study of ancient civilisations.

Using archaeological objects as sources of aDNA is not without obstacles, as the methods for collecting samples are invasive and often damages or even destroys the object, e.g. a tooth, leaving nothing for future generations to appreciate and study. Our discovery provides a whole new approach, as historical material made from clay deriving from ancient Mesopotamia is highly abundant, and the location and time period of the artefacts can be determined through the cuneiform inscriptions on the objects and the knowledge of the archaeological sites where they were discovered. We furthermore encourage future studies to develop methods to obtain samples from the core of bricks or tablets without destroying the objects, leaving only a minimum of visible damage. Extracting samples from the core of the brick would provide insurance that the samples derive from a shielded, and therefore uncontaminated, environment inside the mass of clay.

The continuously accelerating development of sequencing technology has made aDNA analysis more accessible than ever. Both amplicon sequencing of short genetic markers, as well as metagenomic shotgun sequencing are being applied to diverse ancient and degraded materials in order to elucidate the taxonomic composition of our past.

The presented study uses a modified protocol that has previously been applied to materials such as bone, considering that clay samples are porous with high affinity towards nucleic acids. This required a gentle approach to extract the aDNA without degrading it further by applying harsh treatments. The applied method was successful in extracting plant DNA from the samples of a clay brick.

The choice of DNA sequencing approach and bioinformatic analysis also has implications for the results that can be obtained^38^. Shotgun metagenomic sequencing captures short fragments of DNA from all genomic material in a given sample and is theoretically able to capture a more representative picture of the taxonomic content of the analysed material. However, available databases are biased toward the most well-described taxonomic markers and European, North American and East Asian organisms. It may be difficult to provide a precise identification of all shotgun data because of this. In addition, it is not always possible to obtain aDNA samples with sufficient DNA quality to perform this analysis from all materials without rigorous optimisation, such as the highly porous and brittle material from the clay brick used in the present study. Amplicon sequencing focuses on short fragments of well-conserved and well-described genes such as those found in the ribosomal operon or chloroplast and mitochondrial genome. This can produce a precise identification of observed DNA variants, but due to the narrow range of targets compared to the total genomic material available, it may also cause a bias toward the most abundantly observed organisms. The present study shows that amplicon sequencing makes it possible to provide insight into the overall taxonomic groups of plants present in very small fragments (88–421 mg) of clay brick material, but future metagenomic studies may improve identification of individual species as chloroplast DNA is notoriously well-conserved between certain plant families and genera.

After the amplicon sequencing of the aDNA samples was completed, the remaining aDNA extract from sample 2 and 5, those with the most successful amplification and identifications, were used to generate a shotgun metagenome, using the genomic DNA ligation sequencing approach from Oxford Nanopore Technologies. However, as the amount of DNA entering the protocol was already very low (≤ 0.1 ng/µL), due to the expected DNA loss during library preparation, the output of this was limited (approx. 105.000 reads of 50–300 bp in size, with sufficient quality for analysis). Based on this limited data, it was not possible to determine the characteristic deamination DNA damage patterns that is unique to aDNA. However, it was possible to recover short fragments of plant DNA, with identifications matching those of the taxonomic groups found by the amplicon data. For future analysis, it is recommended that sampling procedures and DNA extraction is further optimised to improve yield, which in turn should allow for the verification of ancient DNA molecules using aDNA-targeted softwares, by use of metagenomic DNA analysis. Due to the brittle nature of the clay, it is expected that sampling size or at least depth will likely need to be increased to achieve sufficient DNA yield for robust analysis. We suggest that ancient DNA procedures investigating bones, a similarly porous and brittle material^39^, could potentially provide insight into future aDNA extraction improvements.

With this research we have made the discovery that ancient DNA, effectively protected from contamination inside a mass of clay, can successfully be extracted from a 2,900-year-old clay brick. We detected 34 unique taxonomic groups of plants in our samples. We have demonstrated our methods and encourage future research into this subject, as the scientific potential for this approach is substantial for several academic fields such as ancient genomics, Assyriology and Near Eastern archaeology, climate and biodiversity in historical contexts, and it will lead to a deeper understanding of ancient and lost civilisations.

## Methods

### Sampling of the brick

The initial sampling of the clay brick (museum number 13854) was conducted on the 7th of October 2020, shortly after a new and uncontaminated break had occurred in the clay brick. Four samples were extracted from the new break. The sampling took place in the storage facility housing the brick at the National Museum of Denmark. All samples were collected by T.P.A. T.P.A. wore a mask and latex gloves throughout the process, and the gloves were disinfected with 85% ethanol between each sample extraction. The first four samples were obtained by employing sterile scalpels for each sample, picking or scraping off clay from the surface of the break. The final fifth sample consisted of a lump of dried clay from the boundary between the new and old break, which became loose during handling when the brick was moved. The samples were transferred directly into five sterile Eppendorf tubes. The samples were stored at room temperature and were shielded from sunlight until the process of DNA extraction was initiated. The five pieces of clay weighed 187, 421, 306, 88 and 272 mg, respectively.

### DNA extraction

DNA extractions were performed in a clean room separate from the rest of the molecular laboratory facilities, on a different floor of the building, under sterile conditions. The DNA extraction itself was performed in a laminar airflow bench with HEPA air filtration that was deep cleaned with 10% bleach solution, and UV treated for 1 h prior to beginning the extraction procedure. A sterile medical swab was used to sample all surfaces of the bench, and was analysed alongside the aDNA samples as a control. No personnel except the person performing the DNA extraction was present in the clean room during the work, wearing head-to-knee personal equipment at all times. Samples were only handled inside the UV-bench. All surfaces, tools and personal protection items such as gloves, and sleeve protectors were sterilised regularly with 10% bleach, 70% ethanol and RNAseAWAY (Thermo Fisher Scientific).

All samples were extracted using a combination of two previously published protocols^39,40^. Briefly, samples were covered with 1 mL of lysis buffer (0.45 M EDTA pH 8.0, 0.25 mg·mL^−1^ proteinase K, 0.5% *N*-laurylsarcosyl), and incubated for 1 h at 56 °C with shaking, after which the supernatant was removed, and the solution was stored at − 20 °C overnight. A second lysis step was performed with another 1 mL lysis buffer and incubation for 1 h at 56 °C, followed by overnight at 37 °C with shaking. Samples were centrifuged for 2 min at 13,000×*g*, supernatant was removed, and mixed with the lysate from the first lysis step. Subsequently, 2 mL of 10 mM Tris–EDTA was added, and the lysates were concentrated down to ~ 200 µL using Amicon Ultra 4 (30 kD) columns. DNA was isolated, washed and eluted using MinElute columns (Qiagen), according to manufacturer’s instructions with minor changes^39,40^: Columns were dried for 5 min at room temperature prior to elution using 60 µL EB buffer that was pre-heated to 60 °C. A non-template DNA extraction sample was also processed with the same procedure as an additional quality control. DNA concentration was estimated using Qubit dsDNA High Sensitivity assay kit, and a Qubit 3.0 fluorometer (Thermo Fisher Scientific).

### Amplicon PCR and nanopore sequencing

The PCR amplification procedures were performed in a lab where pre- and post-PCR work are separated, and all procedures were performed under sterile conditions. The flora composition of the aDNA in the clay brick was investigated using a well-described DNA barcode (trnL-gh) for the chloroplast *trnL* gene^41^ and the mitochondrial 12S rRNA gene^42^, the latter of which was amplified primarily as a control. The trnL-gh and 12SV05 DNA barcodes were amplified in quintuplicate 25 µL PCR reactions (1 × PCRBIO Ultra Mix, 400 nM of the forward and reverse primer) with 2 µL template DNA, using the following PCR programme: 95 °C for 2 min, 40 cycles of 95 °C for 15 s, 50 °C (trnL-gh and 12SV05), 72 °C for 60 s, and final elongation at 72 °C for 5 min. Replicate PCR reactions were subsequently merged. All generated amplicons were purified using CleanNGS beads (CleanNA) with a sample:bead ratio of 5:4, and eluted to 20 µL of UV-treated nuclease free water. The DNA concentration was estimated using the Qubit dsDNA High Sensitivity assay kit, and amplicon size was checked using D1000 High Sensitivity ScreenTapes on a TapeStation 2200 (Agilent). In addition, the remaining DNA extracts from samples 2 and 5 were pooled and prepared for metagenomic sequencing using Nanopore sequencing, using the same protocols as the amplicons, but on a separate flow cell.

Samples were barcoded and prepared for Nanopore sequencing in accordance with the manufacturer’s recommendations (PBAC96_9069_v109_revQ_14Aug2019) for amplicon sequencing with PCR barcoding using a Ligation Sequencing kit version 109 (Oxford Nanopore Technologies), and sequencing was performed using a R 9.4.1 flow cell. Sequencing was performed for ~ 16 h.

### Quality control and data validation

Raw sequencing data from the amplicons was basecalled using Guppy v4.2.2 with the R 9.4.1 High Accuracy (HAc) model (https://community.nanoporetech.com). Demultiplexing and adapter removal was performed using Porechop v0.2.3 (https://github.com/rrwick/Porechop), with stringent quality filtering requirements that discarded all reads that did not have both barcodes with a quality score of at least 85. The demultiplexed data was subjected to further quality control through NanoPlot v1.38^43^ and was quality filtered to the expected size range (read length 50–500 bp) with NanoFilt v2.6.0^43^ with a minimum q-score of 10. Then, the reads were aligned using minimap2 v2.17^44^ and polished using Racon v1.3.3^45^, and subsequently clustered into Operational Taxonomic Units (OTUs) at 97% sequence similarity using VSEARCH v2.13.4^46^. Taxonomy was assigned using BLAST^47^ against the nt database (downloaded 08-03-2022), using minimum criteria of minimum query coverage 60%, and maximum e-value 10·e^-10^. Representative sequences of each identified organism were then manually curated with additional blast searches. Taxonomy was assigned conservatively, generally only to family level or above, to limit the risk of false positive identification from short sequences.

The data obtained from the metagenomic sequencing was quality checked and filtered using the softwares Porechop and NanoPlot, as described above for the amplicon data. NanoFilt was used to select all reads with a minimum Q-score of 15, but no size filtering or trimming steps were performed to preserve the short reads that were most likely ancient DNA from the clay brick. The data was then analysed using PMDtools v0.60 (https://github.com/pontussk/PMDtools), using two different methods for detecting aDNA damage patterns. Minimap2 was used to align the data to a human (GCF_000001405.40), wild cabbage (*Brassica oleracea*, GCF_000695525.1) and E.coli K-12 (GCF_000005845.2) reference genome assembly downloaded from refseq at NCBI, as human, cabbage and bacteria were the most likely sources of aDNA present in the samples. The data was scanned for deamination damages, using –deamination, and the alignment was also analysed for C to T mismatches in the 5′ and 3′ ends of the metagenomic reads.

## Data Availability

All data has been made available at the European Nucleotide Archive under project accession number PRJEB57874.
